# Ageing, frailty and resilience in Botswana: rapid ageing, rapid change. Findings from a national working group meeting and literature review

**DOI:** 10.1186/s12919-019-0171-z

**Published:** 2019-11-19

**Authors:** Barbara Lachana Onen, Ciara Harris, Agnieszka Ignatowicz, Justine Davies, Michalis Drouvelis, Andrew Howes, Oathokwa Nkomazana, Churchill Lukwiya Onen, Elizabeth Sapey, Billy Tsima, Daniel Lasserson

**Affiliations:** 1 Royal Free London NHS Foundation Trust, London, UK; 2 Faculty of Medicine, University of Botswana, Gaborone, Botswana; 3 Institute of Applied Health Research, University of Birmingham, Birmingham, UK; 4 Department of Economics, University of Birmingham, Birmingham, UK; 5 School of Computer Science, University of Birmingham, Birmingham, UK; 6 Centre for Chronic Diseases, Gaborone Private Hospital, Gaborone, Botswana; 7 Institute of Inflammation and Ageing, University of Birmingham, Birmingham, UK

**Keywords:** Older people, Ageing, Botswana, Research and policy recommendations

## Abstract

**Background:**

The demography of Botswana is rapidly changing. Successes in tackling communicable diseases and economic development increased life expectancy from 53.7 years in 2006 to 66.8 years in 2016. The prevalence of diseases associated with older age, especially chronic non-communicable diseases including diabetes, hypertension and cerebrovascular disease are suspected to have increased but accurate data are lacking. The country has high youth unemployment and national retirement is at the age of 60, which limits the opportunity to build pensions for prosperity in older age. Changes across health, social care and economic policy are needed to prepare for a future ageing population.

**Methods:**

This article reports on the national working group meeting outputs on issues that face older people, their families, healthcare providers, and policy makers in Botswana. A collaborative working group meeting was convened in Gaborone, Botswana on 25th September 2018 by the University of Botswana and University of Birmingham, UK, to identify key challenges and opportunities for an ageing Batswana population.

**Results:**

There was agreement across diverse stakeholders of a need for effective and rapid policy formation to prepare and protect the future health and economy of an ageing Batswana population with a high burden of NCDs. The main priorities for policy-makers must be social care, poverty reduction and healthcare provision, conducted in an evidence-based manner, as far as practicable. To effectively achieve this, research and high-quality data collection mechanisms are required.

**Conclusions:**

Future policy in Botswana must focus on the challenges that an ageing population brings, and development of health and care system resilience for the demographic change could be a model for healthcare policy across Southern Africa.

## Background

Population ageing, while an achievement in terms of health, social care and economic development, is also a significant global challenge. The provision of universal financial security, healthcare, social care, and the prevention of loneliness after retirement age requires significant planning, insightful policy and infrastructure development. Most high-income countries had over 150 years to adjust to a 10% increase in the proportion of the population aged over 60. Lower and middle-income countries will have less than 20 years to make the same adaptation [1].

The demography of Botswana has changed rapidly over the past 30 years and further population shift is predicted. Life expectancy has risen by 13 years from 2006 to 2016, [2] and the total population of Botswana increased by 344,000 between the 2001 and 2011 censuses [3]. Therefore, although the percentage of the population aged 65 and over has stayed relatively stable at around 5.0%, [3] the actual number of Batswana aged 65 and over is increasing.

Despite rapid economic growth since independence in 1966, [4] there is high youth unemployment. The average age of entering paid employment is approximately 33 years [5] but the standard retirement age is 60, [6] leading to a limited period of time to accumulate personal savings and effectively contribute to the economy. Additionally, fertility rates have reduced in recent years, to 2.73 children per woman in 2016 [7].

Although rates of communicable diseases (CDs), such as tuberculosis and other HIV-related conditions, have declined, they are still prevalent [8]. Furthermore, accompanying ageing and declining poverty, is a rise in non-communicable diseases (NCDs) [9] resulting in a double burden of CDs and NCDs [10].

A recent government policy brief highlighted that there is a window of opportunity until 2050, in which there will be a ‘demographic dividend’ where the working-age population will be large enough to allow accelerated socioeconomic development [5]. Thus, changes across health, social care and economic policy are needed to prepare for a future ageing population. A collaborative working group meeting was convened in Gaborone, Botswana on 25th September 2018 bringing together stakeholders from government, public and private healthcare providers, academia, medical insurance providers, patient groups, traditional leaders and traditional healers. In this report, we set out the key themes with recommendations for researchers and policy makers.

## Methods

The organising of the national working group meeting began in May 2018. As part of the process, invitations to attend the meeting were sent to a unique combination of stakeholders, including care workers, nurses, physicians from medical, psychiatric and surgical specialties, researchers, civil servants, traditional healers and village leaders, and non-government organisations. These stakeholders were identified and selected based on their role and experience of working with the Batswana ageing population.

**Fig. 1:**
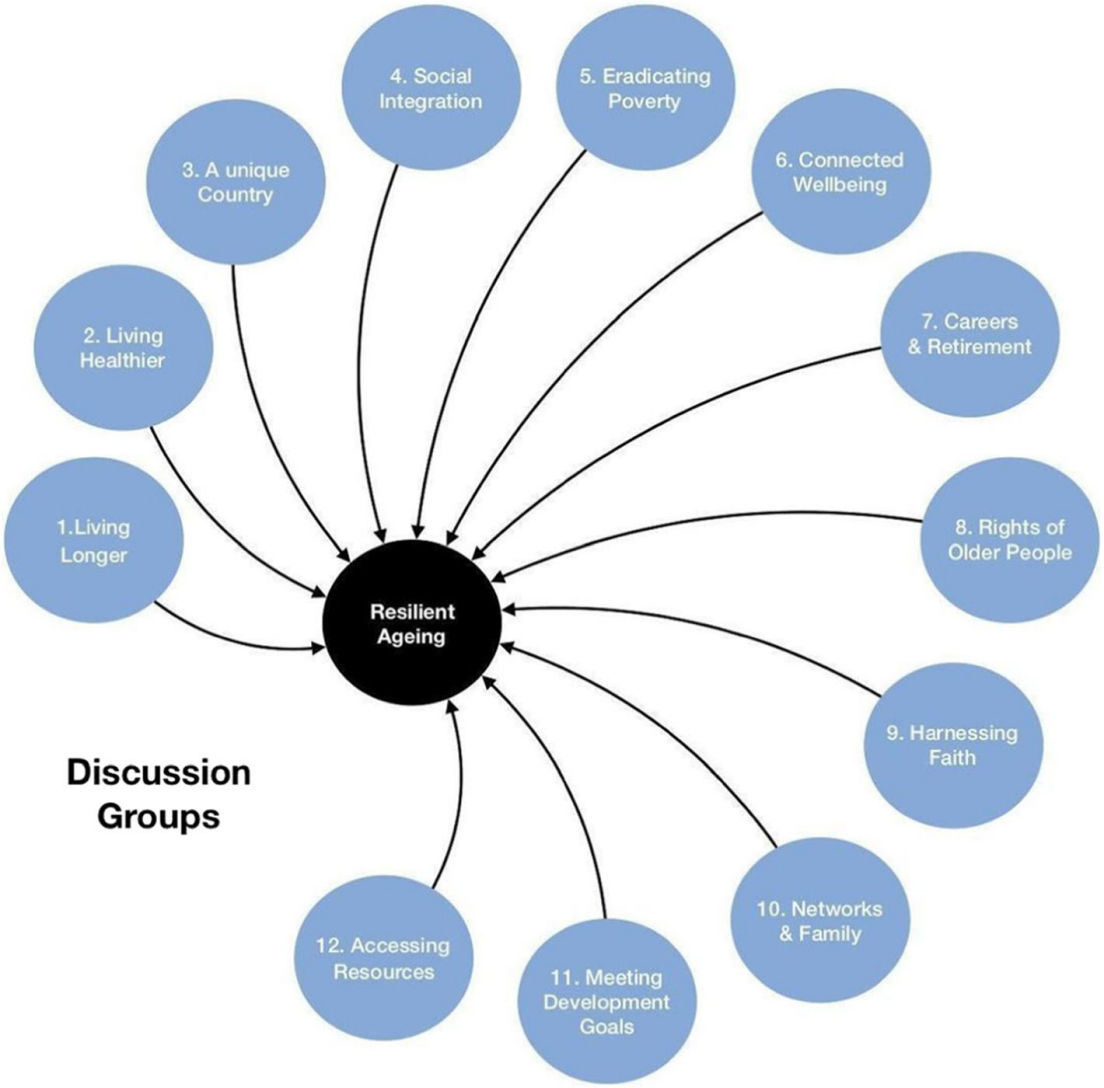
Round-table discussion areas

The meeting began with a panel discussion among local experts to provide focus and context, and was followed by two sets of roundtable discussions with feedback and plenary sessions. The issues discussed in the round tables were selected in advance by stakeholders and reflected areas of importance to older people. The 10 issues discussed at separate round tables are shown in Fig. 1. Notes were taken by all authors of this paper throughout the meeting and used as a basis for drafting the overall consensus statement. The key themes and statements that had been agreed on during the working group meeting were not altered during the drafting of the consensus statement. The end goal of the consensus statement was to provide simple and clear messages on research priorities and recommended policy directions. The consensus statement was initially drafted by the authors of this paper, which was then circulated for review and editing to all the attendees of the meeting. There was no requirement for unanimous agreement, and there was an option for dissenting or minority opinion. The authors of this paper then met to discuss the feedback. Following discussion addressing each of the 10 issues, they then agreed on the summary statement for each issue. The outputs from the roundtables were combined to form five integrated themes of: healthcare delivery; economics - poverty and pensions; social networks and resources; the Botswana Development Goals (BDGs) and data required to inform policy in each area. A literature search was also conducted to identify research and guidelines relevant to the consensus statement. This report was written to include the consensus statement and the synthesis of the published evidence.

## Results

### Healthcare delivery

Cardiovascular diseases (CVDs) are more prevalent in populations with HIV infection due to the effects of HIV and its treatment [11, 12]. Healthcare delivery in response to the HIV epidemic in Botswana represents a significant success [13] with a high level of testing, treatment and viral suppression, [14] close to achieving the global ‘90/90/90 targets’ [15]. However, the complex, interacting burden of physical and mental health with HIV poses substantial health challenges for older citizens, their caregivers and current healthcare infrastructure in Botswana.

As discussed in other Southern African countries, the infrastructure and resources already in place to manage the HIV epidemic could now be leveraged to manage the growing burden of NCDs in Botswana [16]. However, without careful planning, HIV services risk becoming overwhelmed as large numbers of people are currently diagnosed with NCDs with 36% of 50–59 year olds self-reporting hypertension and 7.6% diabetes in 2017 respectively [17]. Additionally, services provided would need to extend to all people with NCDs, not just those with HIV and NCDs. Consideration would need to be given also to how patients with NCDs would perceive and utilise such services, given the remaining stigma around HIV.

Preventative strategies to reduce behavioural traits associated with NCDs should accompany initiatives to increase population-wide knowledge about NCDs. Screening of at-risk groups occurs in many countries, but has not consistently resulted in improved NCD diagnosis and management, [18, 19] and there is as yet no evidence of improved clinical outcomes with screening programs in low and middle income countries. Furthermore, national treatment guidelines for the management of NCDs and their acute presentations, whilst in existence, are thought not to be commonly accessed in Botswana and the lack of NCD expertise across healthcare providers (especially in rural settings) may hamper care delivery of integrated care. Although there is free healthcare and out of pocket expenditure is only 5.26% of total healthcare expenditure, [20] the extent to which necessary healthcare for NCDs in the public sector is available is not known. Further research needs to assess the availability and affordability of treatments for NCDs.

Unfortunately, there is a lack of empirical data on healthcare needs and access of the contemporary older population in Botswana. National surveys including older people are either outdated [21] (for example, Clausen et al. 2007 [21] used data from 1998) or, if more recent, have excluded people over 65 years of age [17]. Whilst it is likely that key challenges for older people include NCDs and barriers to accessing healthcare (both logistically and personally), more data are needed to ensure that solutions are appropriate to needs. In these regards Botswana is similar to many countries within sub-Saharan Africa.

### Economics - poverty and pension

Over the last three decades, a growing economy has contributed to reducing poverty; poverty dropped from 47% in 1993 [22] to 16.3% in 2015/2016, and the proportion of people living on less than $1.90 per day has dropped from 23.4% in 2002/03 to 5.8% in 2015/2016 [23]. However, Botswana remains highly unequal, with a Gini index of 60.5, approaching the highest in the world [24]. Older people may be especially vulnerable to poverty.

There is a considerable gap between labour income and consumption across different age populations [5] due to high youth unemployment, relatively early retirement and low pension provision. The state retirement age in Botswana is 60 years, [6] although some employment sectors allow variation of this, for example Botswana Defence [25] and judicial service [26]. The state pension (430 Pula per month) is available to Batswana citizens from the age of 65 years [27]. This leaves a potential earnings-pension income gap of 5 years for those without an industry-based or private pension provision. The proportion of adults in Botswana who have private/supplemental pension provision is low [28] and the 5-year retirement-pension gap could be impoverishing for some who are reliant on the state pension.

Nevertheless, there are efforts to support the vulnerable, for instance the government has instituted a Destitution Programme based on permanent or transitory poverty with provision of a food basket, valued at 500–600 Pula a month and a cash allowance of 250 Pula for non-food needs [27]. The number of older people aware of, or accessing, this, and its sufficiency, is unknown.

### Social networks and resources

Later-life loneliness and social isolation are areas of concern in Botswana. The rural-urban migration and general weakening of family ties as a source of both physical and social support are contributory factors for loneliness among older people. Traditional societal expectations placed direct responsibility for care of older people on their families, although this has changed more recently in neighbouring countries [29]. As families fragment, older people without family support are more likely to have unmet functional needs [30].

The reciprocal link between physical and psychological status of older people is well recognised [31]. In Botswana, there is a lack of policies and programmes which could mitigate loneliness and social isolation. Private day care centres, hospices and the Church are engaged in services for older adults, but these are not routinely available or accessible. Religion is a central part of Batswana society and faith can represent an important component in tackling social isolation and loneliness of older people. The mechanisms through which faith can support older people include a sense of hope, belonging to a community, reassurance from belief in an afterlife and a mission giving an outward focus of care. Additionally, older people have valued and respected roles within many faiths.

Services to tackle later-life loneliness and social exclusion need to be more widely available and robust if they are to promote healthy ageing, build resilience and support independence. National policies and standards on health and well-being of older people would have to interface with other aspects of the system, such as provision and viability of pension and health insurance schemes. However, it is unclear if the government can logistically and fiscally support sustainable projects for older people, based on appropriate community structures. A comprehensive assessment of the availability of services to counter loneliness and maintain independence is required.

As in other societies, older people may not always be aware of their rights, especially those living in more rural communities or among those who were unemployed or lack formal education. Age is not a protected characteristic in discrimination laws and there are no specific provisions or charters in Batswana law that specifically refer to older citizens. Possible options for older people who wish to raise a concern regarding their rights or legal matters within Botswana include lawyers, police, social workers and the ombudsman, but there may be inequalities in accessing these services.

Physical and digital infrastructure in Botswana is not optimised for the older person. Most buildings lack lifts or ramps to improve accessibility and stairs, paths and pavements are not well maintained. Access to information through the Internet was considered challenging in a country with a small but highly distributed population. The transition from extended families to nuclear families demands a different technological infrastructure to maintain connections. There was a concern that insufficient bandwidth might inhibit uptake of data-driven healthcare applications that can adapt to individual and culture specific needs. A related concern was relatively low adoption of mobile health technologies in older generations [32].

### The Botswana sustainable development goals

Older people are not specifically referred to in the Botswana Sustainable Development Goals (BDGs) [33]. The goals highlighted in the Voluntary National Review were discussed individually [34].

#### BDG1: end poverty in all its forms everywhere

Older people in Botswana are at a high risk of poverty, including extreme poverty, which is defined by The World Bank as being <US$1.90 PPP per person per day in 2015 [35]. For older people with a pension as the sole source of income and for an average month of 30 days, the daily “wage” is US$3.80 PPP (converted using PPP, as per World Bank poverty line calculations). Although this is above the international poverty line, it is below the Upper Middle Income Class Poverty Line of US$5.50 PPP [36].

#### BDG 2: end hunger, achieve food security, improve nutrition and promote sustainable agriculture

There is lack of data to inform the scale of hunger and poor nutrition among older Batswana. The 2014 STEPs study, [37] showed that, among those aged 60–69, 15.4% of men and 5.1% of women were deemed to be “underweight” (body mass index (BMI) of < 18.5 kg/m2). In the same age group, the rates of people deemed to be “over-weight” and “obese” were 34.5 and 65.8% in men and women respectively.

The Integrated Support Programme for Arable Agricultural Development (ISPAAD) promotes arable farming in Botswana, and is primarily utilised by poor households, most of whom fall below the Poverty Datum Line and are farming for subsistence purposes [38]. The largest beneficiary group of ISPAAD comprises people aged 65 and above (at 28%) [38]. The population can access seeds and fertiliser (for farming up to 5 ha in size), organised through agricultural officers who register citizens for the scheme throughout the country. The Government also pays for shared equipment schemes to support harvesting [38]. Potential barriers may include difficulty accessing the offices to register, limitations in physical function and an unfavourable climate. The retention of older, experienced farmers within a wider farming community may have significant benefits and there could be positive intergenerational networking to improve agricultural skills among rural populations.

#### BDG 3: ensure healthy lives and promote well-being for all

The stated goal to prevent “premature mortality from NCDs” generally refers to adults aged up to 69 years [39]. This could be considered an arbitrary and premature cut off given the rapidly ageing population of Botswana, although currently the population over 60 years old in Botswana is still only 6% [40]. There is a risk of impoverishment related to personal healthcare spend but current data are lacking. The 2002–2003 Botswana Household Income and Expenditure Survey described 11 and 7% of households facing catastrophic expenditure at threshold of 20 and 40% respectively [41].

#### BDG 5: achieve gender equality and empower women and girls

Whilst younger women may be empowered, older generations of women may be less so. Of the existing research on gender in Botswana, [42–44] there has not been research on transgenerational gender experience. Furthermore, the World Development Indicators [45], which compiles the international statistics on global development, do not contain any data on the involvement of women in making decisions.

#### BDG 9: industry, innovation, and infrastructure

Infrastructure is currently being built without an assessment of impact on older people. Services centred around older people’s needs are required, particularly for those who live in rural locations. There are no published assessments of current infrastructure development plans and their impact on accessibility for older people.

#### BDG 17: strengthen the means of implementation and revitalise the global partnerships for sustainable development

There is significant utility of South-North, South-South, and North-South partnerships in achieving sustainable development. The 2015 Third International Financing for Development Meeting in Addis Ababa further encouraged such partnership working [46].

Notably, the other development goals should also consider the needs of older people including BDGs 4 (inclusive and equitable quality education); 6 (sustainable management of water and sanitation); 7 (affordable sustainable energy); 8 (sustainable economic growth and decent work); 10 (reduce inequalities); 11 (safe and sustainable cities and communities); 12 (responsible consumption and production); 13 (action on climate change); 14 (sustainable use of seas); 15 (sustainable use of ecosystems on land); and 16 (peaceful and inclusive societies supported by just institutions) [34].

### Data required to inform policy in each area

Good quality data are essential to accurately plan, fund and evaluate programmes and activities, and to monitor the progress towards specific development goals [47]. However, high quality data are often not available in sub-Saharan Africa [48, 49]. Although substantial investments have been made over the last decade to improve the quality and availability of data in Botswana, there remains a need for more comprehensive and timely data about ageing, focusing on the research and policy areas listed above, linked to the BDGs. In light of this, a series of research priorities and policy areas for development were identified.

For identified research priorities see Table 1 and for suggested areas for policy development, see Table 2.

**Table 1:** Identified Research Priorities

Healthcare delivery	1. To map the burden of NCDs to healthcare infrastructure to identify mismatched geographical locations. 2. To model the future economic and healthcare burden of NCDs to inform policy. 3. To assess the perception of NCDs in Botswana in both urban and rural setting and identify potential barriers to screening or treatment. 4. To test screening tools and treatment pathways in Botswana, both for sensitivity and specificity but also feasibility in a rural and urban setting. 5. To test different healthcare delivery models focusing on older age across Botswana.
Economics- poverty and pension	1. Surveys to describe the circumstances of older people in Botswana to encompass levels of income, activities and health in rural and urban settings. 2. Qualitative research to explore relationships and interactions between older and younger people in Botswana and what each group considers the needs of the other to be, comparing rural and urban settings, including social engagement and relevance to their communities (urban versus rural). 3. Determine the prevalence and effects of intergenerational care on social engagement and retirement employment opportunities. 4. Trials of retired employment activities and day centres for retired people (which could include childcare) to determine feasibility and support community engagement.
Social networks and resources	1. To understand the views and experiences of older people, with a clear focus on the key areas that most affect the quality of life experienced by older people and the health and social care they receive. 2. To explore how much older people know about their rights and to assess access to legal services comparing older and younger citizens across rural or urban settings. 3. Qualitative research to determine whether people think age should be specifically referred to in law or if charter for older people should be developed in Botswana. 4. To assess housing for older citizens in urban and rural settings and perceptions about housing 5. To survey public buildings to assess accessibility for older citizens and assess perceptions about public infrastructure.
The Botswana Development Goals	1. Develop mechanisms for data collection that inform on the progress towards meeting SDGs and the extent to which older people benefit as well as younger people in their attainment.

Legend: These form the consensus-agreed research priorities to improve the health, well-being and economic status of older people in Botswana across the discussion themes. The numbers or order do not reflect priority

**Table 2:** Policy areas for development

Healthcare delivery	1. National education programmes and facilities to reduce stigma and modifiable risk factors for NCDs and mental ill-health and identify common symptoms of disease. 2. Developing a Botswana NCD screening strategy including expansion of the Botswana Primary Care Guidelines to include NCDs with training for healthcare workers. 3. New healthcare delivery models including community-based services for NCDs. 4. Develop human resources for the elderly: e.g., geriatric doctors and nurses, social workers.
Economics- poverty and pension	1. Education about retirement planning to future protect the working population. 2. Raising the state pension to levels which support a living wage and matching retirement age to pension provision age. 3. Employers requirement to offer private pension provision. 4. Encourage “civic society” with more volunteering (youth unemployment and older adults) to improve social engagement. 5. Age discrimination laws to protect the older workforce.
Social networks and resources	1. Age as a protected characteristic or a charter for older people built into Botswana’s law. 2. Reform town planning processes to focus on building resilient communities that support people through life including accessibility, mobility and access to communal spaces. 3. Review of, and increase in, support offered to older people in Botswana to build resilience for managing major life changes associated with retirement and ill-health. This could include supervised care, wellness and adult foster homes linked to the Kgosi (traditional chiefs). 4. Invest in data communications infrastructure including health-related personal information technologies. 5. Inclusion of faith groups and NGOs involved in the care of the elderly and other vulnerable people in national events and celebrations but regulate or audit to ensure suitability of service.
The Botswana Development Goals	1. Consider development of specific health goals for older people in Botswana. 2. Consider the policy levers through health, finance and infrastructure through which the SDGs can be achieved with equitable benefit to older people.

Legend: These form the consensus-agreed policy areas for development to improve the health, well-being and economic status of older people in Botswana across the discussion themes. The numbers or order do not reflect priority

## Conclusions

There was agreement across diverse stakeholders of a need for effective and rapid policy formation to prepare and protect the future health and economy of an ageing Batswana population with a high burden of NCDs. The main priorities for policy-makers must be social care, poverty reduction and healthcare provision, conducted in an evidence-based manner, as far as practicable. To effectively achieve this, research and high-quality data collection mechanisms are required. Due to Botswana’s economic position and the recognition by the Batswana government that this is a priority area, Botswana is ideally situated to make progress with both research and policy development, and subsequently inform the provision of care and services to older people in similar sub-Saharan African countries.

## Data Availability

n/a

## Abbreviations

BDGs: Botswana Development Goals
BMI: Body mass index
CDs: Communicable diseases
CVDs: Cardiovascular diseases
ISPAAD: Integrated Support Programme for Arable Agricultural Development
NCDs: Non-communicable diseases
